# Urban Airborne Lead: X-Ray Absorption Spectroscopy Establishes Soil as Dominant Source

**DOI:** 10.1371/journal.pone.0005019

**Published:** 2009-04-02

**Authors:** Nicholas E. Pingitore, Juan W. Clague, Maria A. Amaya, Beata Maciejewska, Jesús J. Reynoso

**Affiliations:** 1 Department of Geological Sciences, The University of Texas at El Paso, El Paso, Texas, United States of America; 2 School of Nursing, The University of Texas at El Paso, El Paso, Texas, United States of America; 3 El Paso, Texas, United States of America; Universidade de Vigo, Spain

## Abstract

**Background:**

Despite the dramatic decrease in airborne lead over the past three decades, there are calls for regulatory limits on this potent pediatric neurotoxin lower even than the new (2008) US Environmental Protection Agency standard. To achieve further decreases in airborne lead, what sources would need to be decreased and what costs would ensue? Our aim was to identify and, if possible, quantify the major species (compounds) of lead in recent ambient airborne particulate matter collected in El Paso, TX, USA.

**Methodology/Principal Findings:**

We used synchrotron-based XAFS (x-ray absorption fine structure) to identify and quantify the major Pb species. XAFS provides molecular-level structural information about a specific element in a bulk sample. Pb-humate is the dominant form of lead in contemporary El Paso air. Pb-humate is a stable, sorbed complex produced exclusively in the humus fraction of Pb-contaminated soils; it also is the major lead species in El Paso soils. Thus such soil must be the dominant source, and its resuspension into the air, the transfer process, providing lead particles to the local air.

**Conclusions/Significance:**

Current industrial and commercial activity apparently is not a major source of airborne lead in El Paso, and presumably other locales that have eliminated such traditional sources as leaded gasoline. Instead, local contaminated soil, legacy of earlier anthropogenic Pb releases, serves as a long-term reservoir that gradually leaks particulate lead to the atmosphere. Given the difficulty and expense of large-scale soil remediation or removal, fugitive soil likely constrains a lower limit for airborne lead levels in many urban settings.

## Introduction

Increasingly strict controls and eventual elimination of lead-based “anti-knock” additives in gasoline are the uncontested cause of the two-orders-of-magnitude decrease in airborne Pb in the USA since the 1970s [1]. A corresponding decrease in blood lead levels of the USA population accompanied this change [2], [3]; similar events occurred in other nations. Nonetheless, the sources and health effects of the lower levels of lead—a potent neurotoxin in children—remaining in “unleaded” air are current topics of scientific and public health investigation [4], [5].

In October, 2008, the US Environmental Protection Agency (EPA) announced their new airborne particulate standard for lead, 0.15 µg Pb m^−3^ air, averaged over rolling 3-calendar-month periods [6]–[8]. This represents an order-of-magnitude decrease from their 3-decades-old standard of 1.5 µg Pb m^−3^ air. Nonetheless, it was reported that independent researchers and some members of the EPA's scientific advisory panel supported stricter limits, as low as 0.02 µg Pb m^−3^ air [8], [9]. Reaching societal decisions on the regulation of airborne lead requires a comprehensive understanding of the sources of airborne lead and the means by which this burden can be lowered most efficaciously. In the era of leaded gasoline, mobile (vehicular) sources represented the dominant input. In developing the new standard, much focus was placed on such point sources as smelters, lead recycling operations, manufacturing, and combustion as well as on the continued use of leaded fuel for aviation piston engines [6]. Interestingly from our perspective, re-entrainment of lead from previously contaminated soils is not featured in this US EPA outreach document [6], although it is addressed in the more comprehensive legislative material [8].

Most conventional approaches to the source issue incorporate one or more of the following: air modeling based on source and receptor data [10]; using lead isotopic compositions to match sources and to model mixing [11]; and direct micro-analysis of individual particles of particulate matter (PM) [12]. For this study, we used synchrotron-based XAFS (x-ray absorption fine structure) to identify and quantify the major Pb species present in airborne PM collected in El Paso, TX, USA. XAFS is a bulk technique that provides structural information at the molecular level about a given element in a sample. Advantages of XAFS over analysis of individual particles by SEM-XRF (scanning electron microscopy x-ray fluorescence), EPMA (electron probe microanalysis), or PIXE (particle induced x-ray emission) include not being limited by the physical size or Pb concentration of individual particles. Advantages over XRD (x-ray diffraction) are orders-of-magnitude lower detection limits and ability to analyze non-crystalline states. Application of XAS to PM had been limited chiefly to source-collected material [13], [14]; advances in instrumentation now allow interrogation of even the minuscule amounts of elements of interest present on typical ambient monitoring filters [15].

## Results

### Data quality

The quality of the XAS spectra of the 10 samples (first 10 listed in Table S1) was noticeably better than that of the remaining 10. The lead content of these best 10 is greater, and often much greater, than that of the remaining 10, with one exception (Tillman Aug 1999). XAS spectral quality mostly reflects the lead concentration in a given sample, but also is affected by sample preparation and presentation, and differences in experimental setup (e.g., detector, beam line, and beam condition).

### Spectral variation within sample set

**Figure 1:**
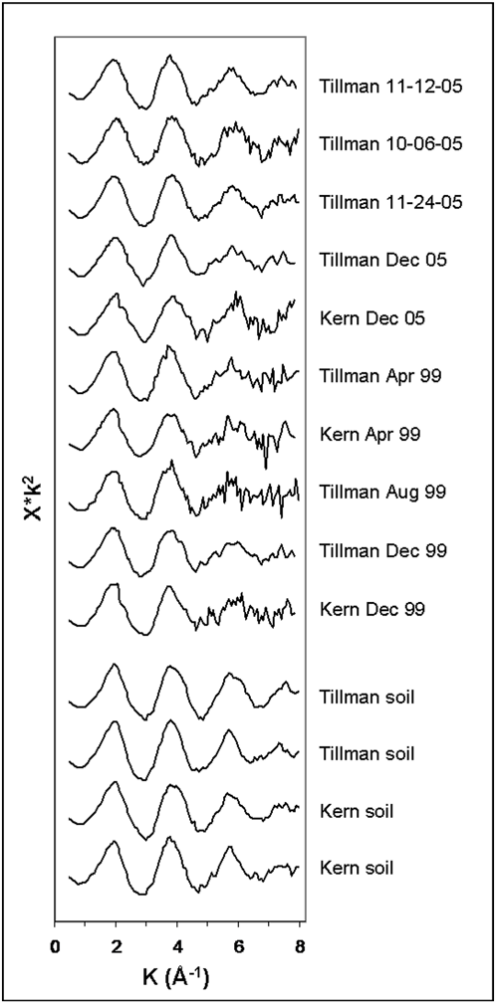
Pb-L_III_ XAFS spectra of PM samples and associated local soils. Spectra are background-subtracted, normalized, *k* ^2^-weighted, and plotted in *k*-space. Even with repeated and stacked scans, the tiny absolute and relative amount of Pb in the PM yielded noisy XAFS, necessitating acquisition of abbreviated spectra. Because an abbreviated region of *k*-space was sampled, we present both the XANES and EXAFS region together as a single XAFS fingerprint for comparison of spectra.

The sample spectra (Figure 1) exhibit the same overall XAFS (x-ray absorption fine structure) pattern, as do the lower quality spectra, not shown. This suggests that the respective PM in the entire sample suite share a single dominant lead compound, or, less likely, two or more compounds in a fixed proportion. Systematic seasonal, geographic, or secular variation in Pb speciation in the El Paso airshed was not evident. Although none of the Northeast sampling station (Figure S1) spectra were of sufficient quality to permit full analysis, lead speciation in these is consistent with the Kern and Tillman spectra. The four soil spectra, taken from the Tillman and Kern neighborhoods, are comparable to the spectra of the air samples, implying similar overall Pb speciation.

### Spectral comparisons of samples and model compounds

**Figure 2:**
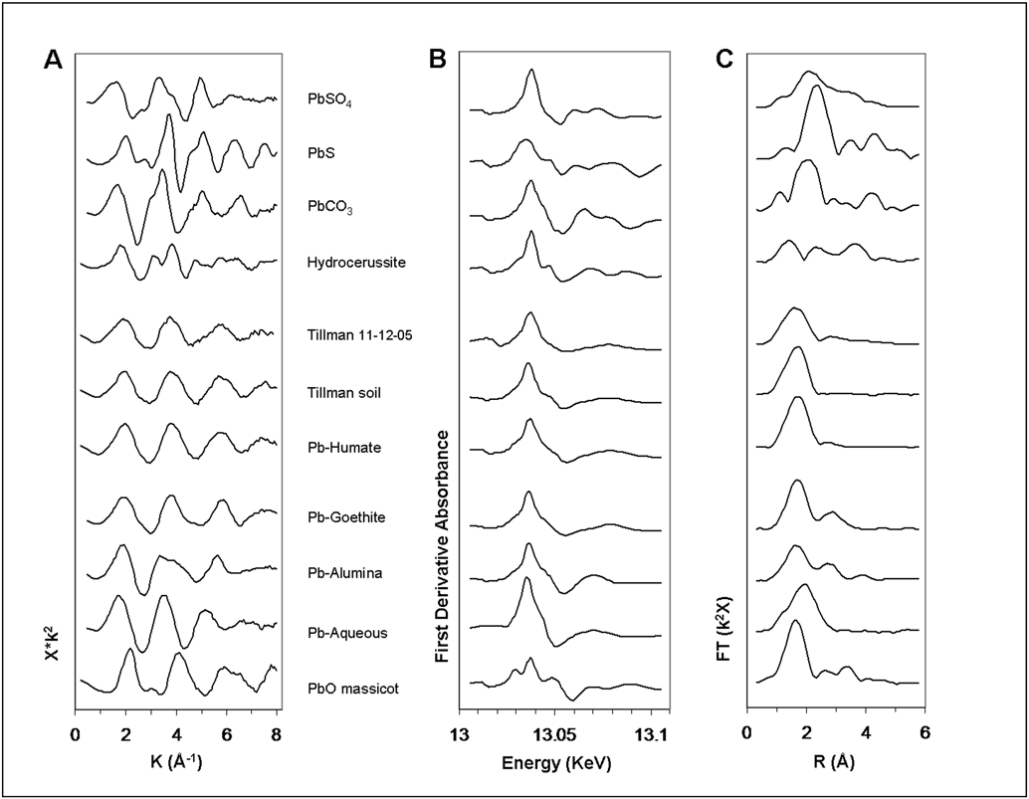
Pb-L_III_ XAFS and derived spectra of model compounds and representative PM and soil. (A) XAFS spectra. Spectra are background-subtracted, normalized, *k* ^2^-weighted, and plotted in *k*-space. (B) Derivatives of Pb-L_III_ XANES spectra of model compounds. Derivatives were taken of spectra that had been background-subtracted, normalized, and *k* ^2^-weighted. (C) Radial distribution functions (RDF) derived from Fourier transforms of Pb-L_III_ XAFS spectra of model compounds. Distances are not corrected for phase shift during photo-electron backscattering. Aqueous Pb spectra adapted from Bargar [30].

Figure 2A presents XAFS spectra of representative model compounds, selected based on their prevalence in natural and environmental settings. The single best fit to the spectra of the air and soil samples is the spectrum of Pb-humate, a stable lead complex with humic acids, associated with the organic fraction of contaminated soils. This suggests that Pb-humate is the dominant species of Pb in the particulate matter trapped on these air filters.

Figure 2B compares the derivatives of the XANES (x-ray absorption near edge structure) region of the model compounds and samples. This region records such chemical information as valence and coordination environment and serves as a species fingerprint. The close match between PM, Pb-humate, and soil is evident.

Fourier transformation of the spectra of the PM and model compounds yields the distances, uncorrected for phase shift at backscatter, from Pb to increasingly distal shells of neighboring atoms (Figure 2C). In the model compounds (PbS is the obvious exception), and presumably in the samples, the first neighbor is O, at close to 2 Å (all distances uncorrected). Peak amplitude of backscatter from shells of neighbor atoms decreases rapidly with distance from the source Pb atoms. The prominent peaks at ∼3.5 to 4.5 Å in most of the standards represent backscatter from a shell containing Pb atoms, which scatter the photoelectrons more efficiently than such low-Z elements as oxygen or carbon. The air PM, as well as Pb-humate, do not exhibit third- or fourth-neighbor Pb-shell peaks. Their second, minor peak represents weak backscatter from the carbon neighbor shell. The PM spectral pattern characterizes complexed or sorbed forms of Pb, e.g., Pb-humate, that lack a space-repetitive (crystalline) structure, as found in lead salts. The peak at ∼3 Å in Pb sorbed to goethite documents backscatter from the ordered Fe shells in that mineral and aids in distinguishing this material from Pb-humate.

### Least-squares fitting of samples with model compounds

**Figure 3:**
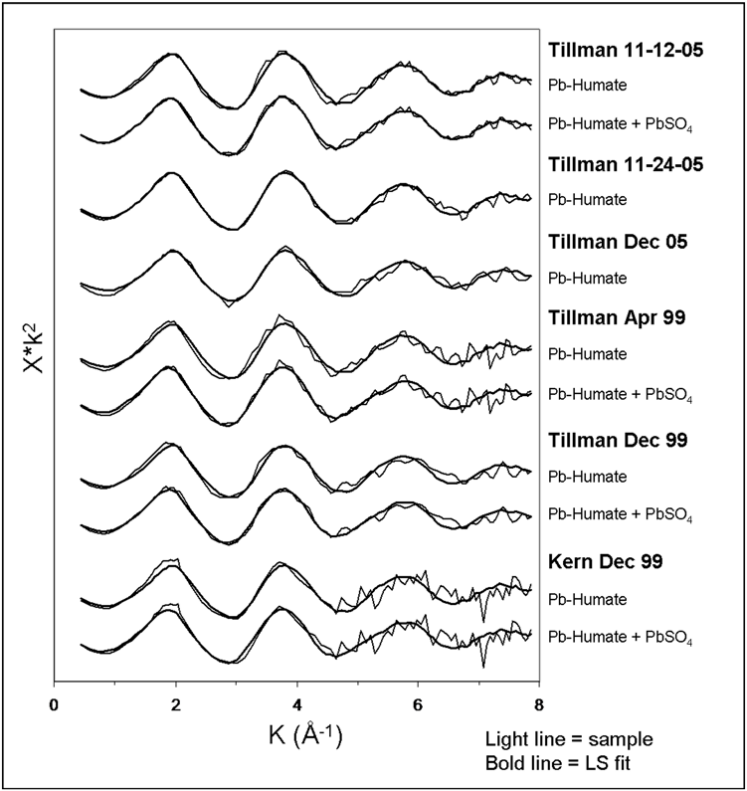
Least-squares fits of XAFS spectra of PM with, successively, Pb-humate and Pb-humate+PbSO_4_. The contribution of PbSO_4_ to the fit in each of 4 cases was approximately 15%; it did not improve the fit appreciably in two cases and was omitted.

Least-squares fits with Pb-humate, and in some cases minor amounts of PbSO_4_, provide a compelling match to the spectra of the PM (Figure 3). Pb-humate dominated the spectra of both the El Paso soil samples reported herein and an additional suite not reported (32 total), with, in cases, the presence of PbSO_4_ or PbO as a secondary component. We conclude that Pb-humate is the major species of lead in our samples of airborne PM, as well as in El Paso soils. Attempts to fit the PM spectra with combinations of two model compounds, not including Pb-humate, were not successful. Although the fit of the PM spectra by Pb-humate is compelling (Figure 3), it is possible that a tested or untested lead species might be present in the PM in significant, but undetected, quantity and still provide a computationally equivalent fit [16]. Because the Fourier transforms did not reveal a significant lead or metal neighbor, any other putative major component would involve sorption of lead on a low-atomic-number host, e.g., certain clay minerals common in soil. Nonetheless, the affinity of lead for soil organic matter is well known and Pb-humate has been identified in soils by other researchers using XAS (see next section). Further, we are unaware of any major non-soil emitter of lead sorbed on a low-atomic-number host. For example, preliminary analysis of XAS spectra of automobile air intake filters and exhaust pipe scrapings indicates significant contribution from a different form of Pb, still unidentified. We presume that this compound is produced from the lead (much of it natural) in the hydrocarbons being combusted by vehicles and released along streets and highways; its spectrum does not resemble that of Pb-humate.

It would prove difficult to identify Pb-humate in airborne particulate matter by such standard single-particle elemental analysis techniques as SEM-XRF, EPMA, or PIXE. The low levels and dispersed nature of the lead in humate complexes is not suited to the detection limits of these instruments. Further, the small absolute and relative abundance of Pb on PM filters by and large precludes confirmation of this result by such alternative conventional analytical techniques as sequential extractions. Nonetheless, the similarity of the spectra of air, Pb-humate, and El Paso soil samples, and the apparent dominance of Pb-humate in those samples, convince us that fugitive soil is the major source of lead in local airborne particulate matter.

## Discussion

### Properties and bioavailability of Pb-humate

It has long been understood that in natural soils Pb has a high affinity for soil organic matter [17], and this element typically resides near the ground surface, due to the concentration of organic matter in that zone [18]. Pb-humate is a common byproduct of the interaction of introduced lead species with the chemical environment of natural soils [17]. Several XAS studies have identified and quantified Pb-humate as a significant component of Pb-contaminated soils [19], [20].

Sposito [21] stressed that carboxyl and phenolic OH groups provide most of the functional group acidity of humic substances (humic and fulvic acids). McBride [17] emphasized the importance of the biphenol (catechol) group, which dissociates to the ligand, phenolate, on which Pb^2+^ forms an inner-sphere complex. Morin et al. [20] and Xia et al. [22] provide structural details and XAS analysis of Pb-humate, and Guo et al. [23] of Pb-POM (particulate organic matter). In these views Pb-humate is a stable chemisorbed species that is not easily mobilized chemically. Mechanically, we expect Pb-humate to be selectively re-entrained to the air from such soil due to its low mass density (in contrast, primary Pb phases, e.g., PbO or PbSO_4_, are much denser than common soil components) and its concentration in the uppermost soil zone.

The bioavailability of respired or ingested (many inhaled particles >10 µm in aerodynamic diameter are swallowed in the process of mucociliary respiratory tract clearance) Pb-humate is unknown. Experimental investigation of soil lead bioavailability apparently has not included Pb-humate or Pb-humate-rich soil [24]. Oral bioavailability from soil typically is considered to be less than that from soluble lead salts. Nonetheless, the apparent importance of Pb-humate in PM suggests the need for quantification of the bioavailability and toxicology of this material.

### Soil as the dominant airborne lead source in Los Angeles and El Paso

In modeling the California South Coast Air Basin (Los Angeles area), Harris and Davidson [10] found that inputs of lead from mobile (chiefly vehicular—raw and refined petroleum contain trace amounts of natural lead) and stationary sources were an order of magnitude less than output of lead from the basin. They estimated the latter from measured values of airborne PM lead and calculated PM deposition rates. The authors hypothesized that resuspension of previously deposited lead could explain the large discrepancy between input and output. Earlier studies also stressed this putative role of resuspension [25], [26].

Our direct demonstration that soil is the primary source of lead in El Paso air provides robust independent evidence of the validity of the resuspension hypothesis, albeit in a different airshed. Harris and Davidson [10] estimated that resuspended soil provided 54,000 kg of the 61,000 kg of lead input (∼90%) to the South Coast Air Basin in 2001. Our least-squares fits (Figure 3) are consistent with this near-total dominance of soil as the source of airborne lead.

Hot dry climates characterize both the El Paso airshed and the California South Coast Air Basin. Resultant atmospheric and soil conditions, and sparse wind-breaking vegetative cover, favor resuspension and transport of soil. The contribution of fugitive soil to the airborne lead load in wetter climates might be lower, although still significant since at least a short dry season is nearly universal.

### A widespread and unrecognized problem?

Soil lead reservoirs in urban cores of older cities may be significant but unrecognized sources of *airborne* lead. For example, Mielke et al. [27] found that soil associated with private residential properties in the urban core of New Orleans averaged ∼700 ppm Pb. The direct dangers (ingestion and hand-to-mouth exposure while playing in yards, and similar exposures when such material enters the household) of such soils to children are obvious and widely recognized. Not so clear is the contribution of those soils to the ambient airborne lead burden, particularly at the neighborhood level. Our results for El Paso suggest that the soil—air lead connection in urban cores may be a significant problem, perhaps overlooked by the current widely dispersed placement of regulatory PM monitors for lead.

### Regulatory issue

As a principal source for airborne lead in urban settings, contaminated soil may set a practical lower limit for future decreases in regulation of airborne lead levels. To identify Pb releases to the air, source apportionment would involve measurement and documentation of lead resuspension from specific parcels of soil, a daunting engineering and legal task. At the next step, soil remediation or removal could prove unrealistic or prohibitively expensive. Data from both Los Angeles and El Paso indicate that meeting the suggested 0.020 µg Pb m^−3^ air limit would involve significant soil remediation [8], [9]. Future recommendations for a lower standard will require careful evaluation of soil resuspension to weigh the societal health benefits against the economic expense.

### Conclusions

X-ray absorption spectroscopy demonstrates that the source of the majority of the lead in PM in El Paso, and presumably in other US cities, is not current anthropogenic output. Instead, local contaminated soil, a legacy of earlier Pb releases, serves as a long-term reservoir that gradually is leaking particulate lead, much in the form of Pb-humate, to the atmosphere. Given the difficulty and expense of large-scale soil remediation or removal, this Pb-humate may establish a practical lower limit for airborne lead levels in many urban settings. Current use of leaded gasoline in many parts of the world contributes to local urban soil reservoirs of lead. This ensures and exacerbates a lingering future problem with airborne lead that will continue for decades after cessation of use of this toxic formulation.

## Materials and Methods

### Samples

We analyzed 20 samples of PM collected on TSP (total suspended particulate) filters at 3 municipal monitoring sites (Figure S1) in El Paso in 1999 and 2005. The El Paso City-County Health and Environmental District, Air Quality Program, supplied the filters from their archived regulatory collection. Although replaced for many purposes by PM_10_ and PM_2.5_ collectors, with nominal aerodynamic diameter upper cutoffs of 10 and 2.5 µm, respectively, TSPs (upper cutoff ∼40 µm) were the US EPA testing method for meeting the old 1.5 µg m^−3^ standard for airborne lead, and they are the backbone for meeting the new standard. Eleven samples were material from individual filters representing standard 24-hour collection periods. The remainder were one-month composites incorporating material from 4 to 5 24-hour filters exposed in sequence at 6-day intervals. This latter material represents time-averaged conditions of air quality. Typical air lead values reflected in our filters are in the range of 0.010 to 0.050 µg Pb m^−3^ air (Table S1).

The Tillman air monitoring station (Figure S1) lies in an old core urban region with elevated soil lead levels related to a legacy of leaded gasoline, lead-based paint, and an ore smelter mothballed (closed, but capable of being re-opened) for the last decade [28]. It samples the air of the downtown business, commerce, and transportation (state and interstate highways, 2 international bridges, and major railroad yard) center. The Kern station is in a old residential neighborhood approximately 2 km north-west of Tillman and 2 km east from the old smelter. The Northeast station is approximately 10 km to the northeast, in a residential and light commercial district (Figure S1). Lead masses on filters from the Northeast station typically are several-fold lower than those at the Kern or Tillman stations. Winter samples usually have the highest lead levels due to trapping of PM by inversions; summer levels are the lowest.

### Sample preparation

A technique for presentation of the PM to the synchrotron x-ray beam for analysis required a compromise among several constraints. Dry mechanical removal of the PM from the filter results in loss of the finest material to the air; wet removal could alter the speciation of the lead in reaction with the carrier fluid. Exposure of the PM *in situ* on the filter avoids these issues, but introduces two problems: the lead blank in the filter itself (see next section) and increased beam scatter from the filter mass, which contributes to the limited total counting rate of the solid-state x-ray detector.

We settled on mechanical stripping of the surface of the filter by gentle manipulation with a clean razor blade (no Pb detected in blade by XRF). We estimate from appropriate before-and-after weighings that we typically removed from 5 to 15% of the mass of the filter in this process. Working on a clean, white background indicated that mechanical loss of the typically dark PM during this process was minimal. The process appeared to remove virtually all of the PM: the remaining filter was visually and chemically (XRF) indistinct from a blank.

A typical sample comprised scrapings from ∼20 cm^2^ of filter top; in some cases we were limited to less by the amount of filter available. The material was held in XRF-grade Mylar™ PET film, or supported directly by Kapton™ polyimide tape placed on the sample holder outside the beam footprint. For this study, we estimate that the beam interrogated between 100 ng and 2 µg of lead in the different PM samples, varying with the mass of material used and its lead content.

### Filter blank

PM was collected on Whatman EPM 2000 glass fiber filters, purpose-designed for the US EPA ambient air monitoring program. For acid extraction of the lead (that does not digest the filter) and subsequent atomic absorption analysis, the specified lead blank is quite low, 0.25 µg Pb per 8″×10″ filter (203×254 mm). The US EPA mentions a typical absolute lead content for such filters of 75 µg per filter (typical mass of filter = 4.5 g) [29]. Our XRF blank analysis by standard additions yielded a similar result for absolute lead content. Although the blank is trivial for measurement of lead mass in PM by EPA protocol, it can affect our XAS analysis since the lead within the glass fibers of the filter, in addition to the lead in the PM, is exposed to the synchrotron x-ray beam.

Not surprisingly, our exposure of a blank glass fiber filter of ∼15 ppm Pb yielded a poor XAFS spectrum. Low spectral quality did not permit identification of the actual form of this lead, which presumably is bonded randomly to oxygen within the glass. The spectrum did suggest a short Pb—O distance, similar to that in PbO (massicot). This blank made a noticeable contribution to the spectra of our lowest-quality PM samples. The major “beat” peaks of these sample spectra are displaced toward higher energy, systematically with decreasing lead content and spectral quality. Least-squares fits are consistent with a Pb contribution from the filter approaching 50% in the worst cases. Nonetheless, such samples still include a Pb-humate contribution of 50% or more. Thus even these questionable samples support our conclusions concerning the importance of Pb-humate in PM.

The blank filter contribution is of only minor concern in our higher-quality spectra. If the sample scrapings that we exposed to the beam included 5% of the mass of the underlying filter, for our sample with the highest measured lead loading (Tillman 11-24-05, Table S1) the lead from the filter itself would represent about 3% of the total XAFS signal. For the sample with the lowest measured seasonal lead content (Northeast Aug 99, Table S1), the lead from the filter itself would represent about 21% of the total XAFS signal. The Tillman and Northeast figures become 6% and 35% if 10% of the filter mass or thickness was included with the PM, and 9% and 45% if 15% of the filter was included. For the next-to-lowest measured seasonal lead in a high-quality sample (Tillman Apr 99, Table S1), the values are 6%, 10%, and 15%.

It is evident from the spectra and independently measured or estimated lead content that the spectra chosen for detailed analysis are unaffected or marginally affected by the blank. Only trivial (5–10%) amounts of the blank spectrum could be accommodated in least-squares fits to only several of the chosen spectra. As a further check, we also analyzed several PM_10_ samples taken on high-purity Teflon™ media filters. Mixes of Pb-humate and PbSO_4_ provided excellent least-squares fits to these spectra, with no indication of the presence of our TSP blank compound. We did not detect any lead by XRF on a blank Teflon™ filter. It should, of course, be emphasized that XAS analysis of mixtures typically cannot discern minor phases and least-squares spectral fits are subject to significant margins of error [16].

### Standards—Model Compounds

We analyzed 36 model compounds along with the samples at the synchrotron. These model compounds then served to identify and quantify the composition of the lead in the particulate matter air samples. These were: PbO (massicot); PbO (litharge); PbCO_3_; PbSO_4_; Pb_3_(CO_3_)_2_(OH)_2_ hydrocerussite; PbS; PbCrO_4_; Pb_3_O_4_; PbO_2_; PbCl_2_; PbBr_2_; PbPaint (10 samples of lead-based paints from El Paso houses and antique cans); Pb-humate (5 varieties; hyphen indicates lead sorbed on material); Pb-goethite; Pb-alumina; Pb-silica; Pb-apatite; Pb-soil (3 varieties); Pb_aqueous_; NIST2584 (National Institute of Standards and Technology reference material: indoor dust with ∼1% lead); NIST 2587 (soil with lead from paint, ∼3000 ppm). Pb^2+^ was used to form sorption complexes.

### Standards—Literature

For comparison with our samples, and our standards, we reviewed the XAS spectra of a total of 42 model lead compounds from 13 studies culled from the literature [19], [20], [22], [23], [30]–[38]. We paid special attention to compounds that were potential contributors to airborne PM or those whose spectra appeared similar to our samples. When needed for careful comparison, if the raw spectral data were not given, we digitized the published spectra to permit such manipulations as least-squares fits to our samples. The comparison spectra included: PbSO_4_; PbS; Pb(NO_3_)_2_; Pb(C_2_H_5_)_4_; PbCO_3_; PbSiO_3_; PbCrO_4_; PbO_2_; PbO (litharge); PbO (massicot); Pb(OH)_2_; PbF_2_; PbCl_2_; PbI_2_; Pb_(aq)_; Pb citrate; Pb salicylate; Pb catechol; Pb benzoate; Pb phthalate; Pb(CH_3_COO)_2_; Pb(CH_3_COO)_4_; Pb(CH_3_)_4_; Pb(Fe_3_(SO_4_)_2_(OH)_6_)_2_; Pb(SO_4_)(CO_3_)_2_(OH)_2_; Pb_3_(CO_3_)_2_(OH)_2_; Pb_5_(VO_4_)_3_Cl; Pb_5_(PO_4_)_3_Cl; PbAl_3_H(PO_4_)_2_(OH)_6_; Pb_2_Fe_11_O_19-8_; PbFe_12_O_19_; PbFe_4_O_7_; β-Pb_6_O(OH)_6_(ClO_4_)_4_·H_2_O; PbOHCl; Pb(II)-goethite (*Pb(II)-xxxxx* indicates Pb^2+^ sorbed on that material; authors used various abbreviation conventions); Pb-humate; Pb(II)-birnessite; Pb(II-hematite; Pb(II)-α-Al_2_O_3_; Pb(II)-particulate organic matter; Pb(II)-amorphous silica; Pb(II)-ferrihydrite. Spectra of a given species often were available from several authors, allowing us to consider spectral variation inherent in the XAFS experimental technique.

### Standards—Pb-humate

We produced Pb-humate standards by spiking reagent-grade humic acid with aqueous solutions of lead chloride. We also produced similar standards by spiking a commercial potting soil, local low-Pb soils, commercial “pure” sphagnum moss, and tea leaves (particulate organic matter, or POM). The resultant spectra for the humic acid, potting soil, local soil, and one brand of tea were nearly identical; a second brand of tea and the moss spectra deviated slightly from the rest. Our Pb-humate and related spectra correspond closely to those reported in the literature [20], [22], [23], [35].

### Data collection

XAFS data were collected on beam lines 7-3, 10-2, and 11-2 at the Stanford Synchrotron Radiation Lightsource at typical conditions of 3 GeV field and 80–100 mA current, using Si(220) water- or liquid-nitrogen-cooled monochromator crystals. We examined the Pb-L_III_ absorption edge at ambient temperature and pressure in fluorescence mode using a 13- or 30-element Ge detector, typically with a Se 3 or 6 filter and Soller slits to reduce scattered radiation. Energy was calibrated according to the first inflection of a Pb-metal foil standard (13.035 keV) run in transmission mode between I_1_ and I_2_. Data were recorded via XAS-Collect. The x-ray beam interrogated the tiny mass (ng to µg range) of lead within the sample matrix of filter material and other PM components, including metals. We stacked repetitive scans to improve data quality. Half of the samples yielded spectra that could be interpreted by standard XAS data analysis techniques.

### Data reduction

XAFS spectra were extracted from averaged absorption spectra by pre-edge subtraction followed by spline fitting and were k^2^-weighted to enhance damped scattering oscillations. The radial distribution function (RDF) was obtained by Fourier transforming the EXAFS k^2^-weighted spectra to real space using a Bessel window function. RDFs are not corrected for atomic pair phase shift. We used WinXAS version 3.1 and SixPACK version 0.61 for the data reduction.

### Data interpretation strategy

The low concentrations of lead in our PM did not permit efficient data collection beyond ∼10 k, with spectral degradation typically occurring beyond 6 k. Therefore we did our k-space least-squares matches over the entire spectrum (EXAFS+XANES), rather than following the conventional approach of using just the shorter EXAFS region. This allowed us to match, using the fingerprint analogy, more “ridges” between the samples and the suspect model compounds. We then explored the XANES region separately via the plots of the derivatives of the XANES. Similarly, the EXAFS region was the sole contributor to the Fourier transform analysis of near-neighbor distances.

## Supporting Information

Table S1 Particulate matter air filter samples and their lead contents.
(0.05 MB DOC)

Figure S1 Map of El Paso County and TSP air monitoring stations.
(0.46 MB TIF)
