# Supplementary material for: Urban Airborne Lead: X-Ray Absorption Spectroscopy Establishes Soil as Dominant Source
Source: PLoS One. 2009 Apr 2;4(4):e5019. doi: 10.1371/journal.pone.0005019 (PMC2659775; doi:10.1371/journal.pone.0005019)
Supplement: Table S1 — Particulate matter air filter samples and their lead contents. (0.05 MB DOC) [file pone.0005019.s001.doc]

| Sample ID**^a^**  (Station and Date) | # of days  in sample**^b^** | Pb on filter**^c^**  ng cm^-2^ | Pb in air**^d^**  ng m^-3^ |
| --- | --- | --- | --- |
| Tiillman Apr 99 | 4 | 120 | 31 |
| Tillman Aug 99 | 4 | 69 | 17 |
| Tillman Dec 99 | 4 | 180 | 46 |
| Kern Apr 99 | 4 | 150 | 39 |
| Kern Dec 99 | 4 | 140 | 36 |
| Tillman 11-06-05 | 1 | --- | --- |
| Tillman 11-12-05 | 1 | 210 | 53 |
| Tillman 11-24-05 | 1 | 230 | 57 |
| Tillman Dec 05 | 4 | 160 | 40 |
| Kern Dec 05 | 5 | --- | --- |
|  |  |  |  |
| Northeast Apr 99 | 4 | 36 | 9 |
| Northeast Aug 99 | 4 | 27 | 7 |
| Northeast Dec 99 | 5 | 41 | 10 |
| Kern Aug 99 | 4 | 78 | 19 |
| Tillman 09-19-05 | 1 | --- | --- |
| Kern Jul 05 | 4 | --- | --- |
| Kern Aug 05 | 4 | 40 | 10 |
| Kern 09-19-05 | 1 | --- | --- |
| Kern 10-12-05 | 1 | 51 | 13 |
| Kern 11-24-05 | 1 | 99 | 25 |

**^a^**The first 10 samples were included in the XAS data analysis; the last 10 were not.

**^b^**Samples comprised material from either a single 24-hour filter or a composite of equal areas of the 4 or 5 24-hour filters available from a single month. ICP-MS analyses of Pb in single samples represent that sample, as do data for Tillman Dec 99, Tillman Dec 05, and Kern Aug 05. Pb concentration data for the remaining one-month samples are 4-month seasonal averages that include the 1.5 months before and after the month in question. This seasonal compromise and missing data result from lack of sufficient sample to permit destructive sample digestion for ICP-MS analysis.

**^c^**Where no value is given, there was insufficient sample material to permit destructive testing for lead content by ICP-MS (inductively coupled plasma mass spectrometry).

**^d^**Pb ng m^-3^ in ambient air is derived from Pb ng cm^-2^ on filters by consideration of air flow rate through filter, filter area, and total exposure time.
