# Supplementary figures and images for: Urban Airborne Lead: X-Ray Absorption Spectroscopy Establishes Soil as Dominant Source
Source: PLoS One. 2009 Apr 2;4(4):e5019. doi: 10.1371/journal.pone.0005019 (PMC2659775; doi:10.1371/journal.pone.0005019)

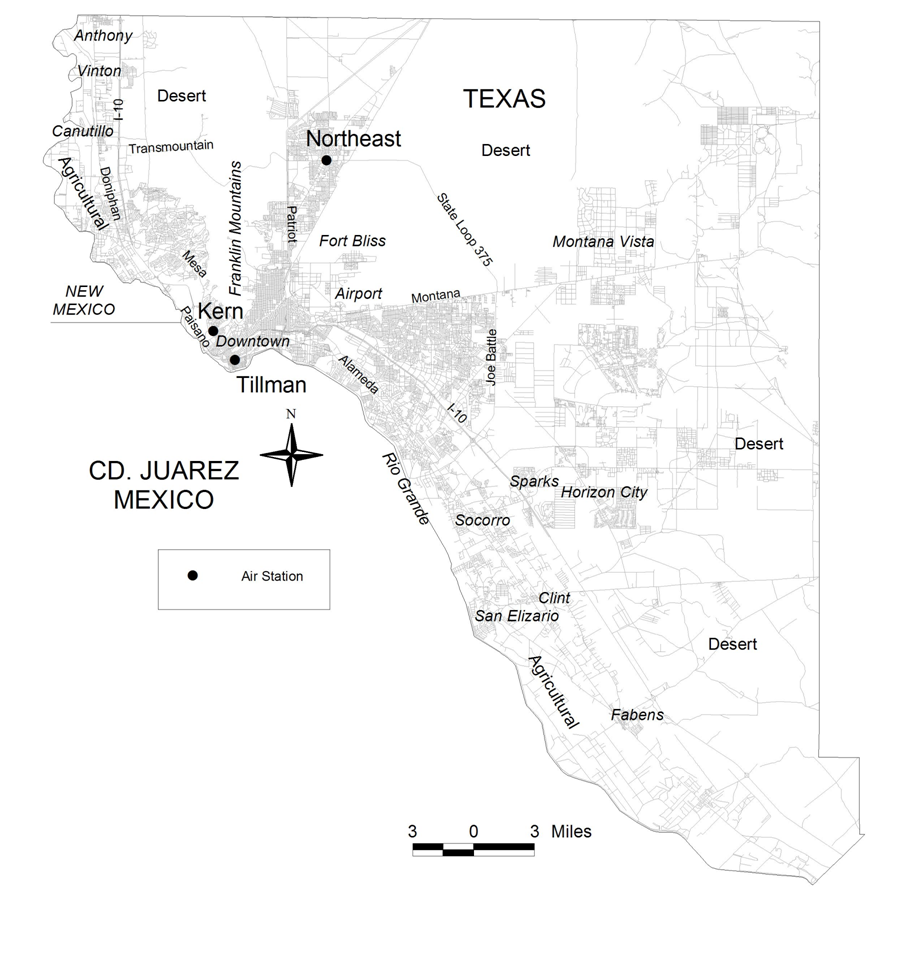

Supplement: Figure S1 — Map of El Paso County and TSP air monitoring stations. (0.46 MB TIF) [file pone.0005019.s002.tif]
